# Biological and Prognostic Significance of the Morphological Types and Vascular Patterns in Colorectal Liver Metastases (CRLM): Looking Beyond the Tumor Margin

**DOI:** 10.1097/MD.0000000000002924

**Published:** 2016-03-03

**Authors:** Pulathis N. Siriwardana, Tu Vinh Luong, Jennifer Watkins, Helen Turley, Mohamed Ghazaley, Kevin Gatter, Adrian L. Harris, Daniel Hochhauser, Brian R. Davidson

**Affiliations:** From the Hepatopancreatobiliary and Liver Transplant Surgery Unit, University Department of Surgery, Royal Free London NHS Foundation Trust (MG) and University College London Medical School (PNS, BRD); Department of Cellular Pathology, Royal Free London NHS Foundation Trust (TVL, JW); Department of Oncology, UCL Cancer Institute (DH), London, UK; Nuffield Department of Clinical Laboratory Sciences, Tumor Pathology Group (HT) and Nuffield Division of Clinical Laboratory Sciences, Department of Medicine (KG), John Radcliffe Hospital, University of Oxford; and Department of Oncology, Weatherall Institute of Molecular Medicine, John Radcliffe Hospital, Oxford University (ALH), Oxford, UK.

## Abstract

Patients with encapsulated colorectal liver metastases (CRLM) have a better prognosis than those without a capsule. The reason for the encapsulation is unknown. Hypoxia inducible factor-1α (HIF-1α) increases tumor angiogenesis and tumor tissue expression is associated with reduced survival. Our aim was to determine whether the good prognosis of encapsulated CRLM is associated with reduced HIF-1α expression by the cancer.

The study selected only patients who had not undergone neoadjuvant chemotherapy prior to a potentially curative hepatectomy for CRLM. From 30 selected patients, serial sections were cut from a single randomly selected metastasis. Morphology was assessed following H&E staining. Tumor hypoxia, vascular endothelial growth factor (VEGF), proliferation, and microvascular density (MVD) were assessed by immunostaining for HIF-1α and carbonic anhydrase-9 (CA-9), VEGF, Ki67, and cluster of differentiation-31, respectively. MVD was calculated in the vascular hot spots. Pathology was reported without clinical outcome information. Actual long-term survival was recorded.

Thirteen (43%) of the cancers were encapsulated CRLM containing glands which were large, complex, and cribriform. Thirteen (43%) were infiltrative CRLM and their glands were small, closely packed, and rounded with vessels in the interglandular fibrous tissue with no capsule; 3 (10%) had a mixed picture. Encapsulated CRLM had a higher expression of HIF-1α (58% vs 8%, *P* = 0.03), CA-9 (42% vs 0%, *P* = 0.04), and VEGF (92% vs 25%, *P* = 0.02). MVD was lower in the encapsulated CRLM group (37 mm^2^ vs 143 mm^2^, *P* < 0.001). The median follow-up was 115 months. The encapsulated CRLM group had a better overall and 5-year survival (relative hazard: 0.58, *P* = 0.057 and hazard ratio: 0.52, *P* = 0.044).

There are 2 main morphological appearances of CRLM which have very different long-term survival following liver resection surgery. The morphology is associated with differences in expression of HIF-1α, CA-9, VEGF, and angiogenesis.

## INTRODUCTION

Development of liver metastases marks an ominous event in the natural history of colorectal cancer (CRC). Historical data suggest that the median survival of patients with colorectal liver metastases (CRLM) without treatment is 5 months.^1^ Surgical resection is the only curative treatment for CRLM. Unfortunately, only 20% of patients are suitable for curative resection at the time of diagnosis.^2^ For those not amenable to resection at time of presentation, down-staging chemotherapy aims to facilitate future resection. Following potentially curative resection for CRLM, about 60% develop recurrences and 80% of these patients are not amenable to further resection.^3^

Identification of prognostic factors in patients with CRLM helps to determine likely survival for an individual patient and allows high-risk patients to be considered for clinical trials. The prognostic markers currently used include serum CEA and CA19-9 levels and the histology of the resected liver metastases. Poor prognostic factors include multiple metastases, poorly differentiated cancers, and the presence of microscopic vascular invasion around the tumor.^4^

An encapsulated form of CRLM was first reported over 15 years ago.^5^ Subsequent studies have shown this variant in 42% to 61% of CRC liver metastases and that it is associated with a significantly better prognosis.^5–7^ The reason for the encapsulation is unknown.

Vermeulen et al^8^ classified CRLM into 3 different types: desmoplastic, pushing, and replacing, with distinct outcomes, on the basis of the growth pattern at the tumor-parenchymal interface. However, these studies, which classified the tumor morphology of CRLM, assessed only the CRLM-liver interface and the invasive front of the metastasis. The morphology of the CRLM–liver interface might be related to the intratumoral morphology.

Tumor morphology based on the degree of differentiation or grade of the tumor has not been found to be a useful prognostic factor in patients undergoing resection of CRLM. This difference between the prognostic significance of tumor encapsulation and lack of prognostic significance of tumor differentiation would suggest that these 2 processes are independent. To the best of our knowledge, the intratumoral histological pattern and the intratumoral vascular pattern of resected CRLM have not previously been described in detail nor have this been correlated with patient outcome.

The effect of local hypoxia on tumor morphology and viability is a major area of research interest as it could lead to development of novel therapies. Hypoxia in resected tissues is generally detected by immunohistochemistry. Van Laarhoven et al found considerable intrametastatic variation of hypoxia in CRLM and this variability was also considerably different between patients.^9^ Hypoxia inducible factor-1 alpha (HIF-1α)^10^ and carbonic anhydrase 9 (CA-9) are 2 key factors induced by cellular hypoxia.^11^ HIF-1α is also induced by nonhypoxic stimuli.^12^ In CRLM, overexpression of HIF-1α is an independent risk factor for recurrent disease.^13^ It activates the transcription of genes which code for more than 300 downstream proteins.^14^ These proteins are involved in glucose metabolism, angiogenesis, cell proliferation, cell survival and invasion, epithelial mesenchymal transfer stem cell growth, and drug resistance.^15^ HIF-1α also activates the transcription of vascular endothelial growth factor (VEGF) which is 1 of the most important factors promoting angiogenesis, which represents a key event in the process of invasion and metastasis.^16^

The rate of tumor cell proliferation can be assessed by detecting fraction of nuclei expressing Ki67.^17^ Cluster of differentiation 31 (CD31) is normally, but not exclusively, expressed in endothelial cells and is a marker used to detect of microvessels.^18^

The aims of this study were to assess whether resected CRLM could be classified into morphological types, based not only on the morphology of the tumor-liver parenchymal interface but also on the morphology of the glands and vascular pattern of the metastases; whether these morphological types of CRLM have a prognostic significance; and whether the morphology may be associated with different levels of expression of hypoxic factors (HIF-1α and CA-9) and VEGF, which influence tumor angiogenesis and tumor proliferation.

Chemotherapy is known to cause areas of intratumoral necrosis within the CRLM and morphological changes to the normal liver parenchyma. Rubbia-Brandt et al conducted a multicenter study that showed the development of perisinusoidal and veno-occlusive fibrosis in patients who have had systemic neoadjuvant chemotherapy for CRLM. These changes were not seen in the chemo naïve control group.^19^ Therefore, in order to exclude the potential confounding effects of chemotherapy on the morphology on CRLM and surrounding liver, a chemotherapy naive group of patients were selected for the study.

## METHODS

### Patients and Specimens

We searched for patients who had not received neoadjuvant chemotherapy for CRLM prior to undergoing hepatic resection at the Royal Free Hospital NHS Foundation Trust over the period 1998 to 2008 to allow adequate follow up to analyze actual long-term outcome. Formalin-fixed, paraffin-embedded hepatectomy specimens of 30 patients with CRLM were retrieved using the pathology database. Informed written consent for research was obtained from all patients (REC ref 5472/6743). Clinical and mortality data were collected from the referring center and the patients’ General Practitioners’ records and the Thames Cancer Registry, respectively, and entered into a secure database (RFH NHS Foundation Trust). Demographic features of the patients, characteristics of primary cancer such as site, stage, differentiation and treatment with chemotherapy, and characteristics of the CRLM such as temporal relationship to primary CRC, differentiation, number and size of metastases, and completeness of resection margin were obtained. Based on the number and size of the CRLM in the hepatectomy specimen, each patient was classified into low, moderate, and high tumor burden groups as described before: low (≤3 metastases and/or ≤3 cm); moderate (4–7 metastases and/or >3–5 cm); and high (≥8 metastases and/or >5 cm).^20^

The tissue block which contained the largest tumor to normal parenchyma interface was selected for each patient. In case of multiple metastases, the metastasis for analysis was selected randomly by an author (MG) who was not aware of the clinicopathological details of the patient. Four-micrometer consecutive sections were obtained for hemotoxylin and eosin (H&E) staining and immunohistochemistry.

### Histology and Immunohistochemistry

The histology of the liver–CRLM interface and the CRLM glandular structures were studied in H&E stained slides independently by 2 experienced liver pathologists (TVL and JW) who were unaware of the clinical outcome of the patients.

Immunohistochemistry was performed in liaison and under guidance of ALH, KG, and HT (co-authors), at the Nuffield Division of Clinical Laboratory Sciences, University of Oxford, with considerable prior experience of assessing tumor tissue hypoxia. Previously described protocols according to manufacturer's instructions were used for immunohistochemistry. The dilution of each antibody which would give the optimum staining was determined by control optimization experiments. In brief, sections were deparaffinized, rehydrated, and antigen retrieved by pressure cooking for 2 min at 125°C while being immersed in the specific antigen retrieval solution for each antibody.

Background staining was blocked using 2 to 3 drops of 2.5% normal horse serum (Vector laboratories) for 20 min. Primary antibody (100 μL) was applied and left at the appropriate temperature and time in a humidified chamber. A postprimary reagent (Novocastra postprimary reagent—Leica Microsystems) was applied for 30 min in HIF-1α, VEGF, and CD31 protocols. The secondary antibody was applied and left in room temperature. Sections were placed in a phosphate-buffer saline bath for 5 min between all steps.

Table 1 shows a summary of the antigen retrieval method, primary and secondary antibodies, and incubation times and temperatures used to assess expression of HIF-1α, CA-9, VEGF, CD31, and Ki67 in CRLM. All primary antibodies were diluted in Roswell Park Memorial Institute solution with 10% fetal calf serum and azide. Diaminobenzidine tetrahydrochloride diluted 1:20 with horseradish peroxidase substrate buffer was used as the chromogen for visualization. The positive controls, for HIF-1α and CA-9, were from formalin-fixed paraffin pellets made from cells grown under hypoxia and, for CD31, Ki67, and VEGF, were sections of tonsil previously demonstrated as being positive. Sections stained without the respective primary antibody were used as negative controls.

**TABLE 1:**
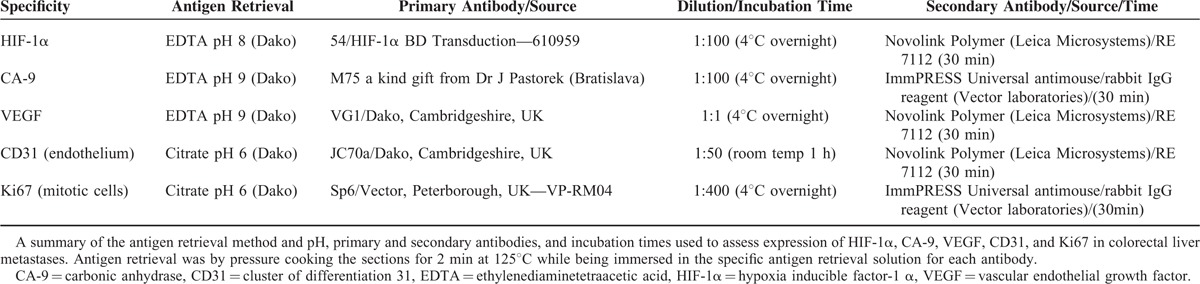
Details of the Immunohistochemistry Staining Protocol

### Scoring and Assessing MVD

**FIGURE 1:**
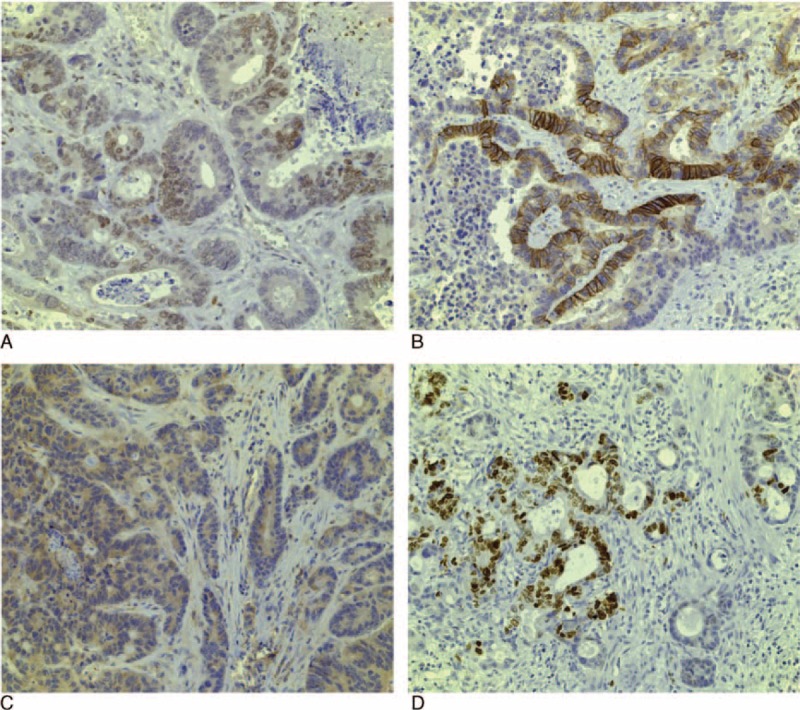
Typical examples of immunohistochemistry staining characteristics of colorectal cancer liver metastasis cells. (A) Hypoxia inducible factor 1 alpha (HIF-1α) ×20: predominantly nuclear with lesser degree of cytoplasmic staining (in cells with high expression). (B) Carbonic anhydrase (CA-9) ×20: predominantly membranous with a lesser degree of cytoplasmic staining. (C) Vascular endothelial growth factor (VEGF) ×20: predominantly cytoplasmic staining. (D) Ki-67 ×20: nuclear staining.

The staining characteristics of the CRLM sections are shown in Figure 1. Previously published immunohistochemistry scoring methods were used to quantify the expression of HIF-1α, CA-9, Ki67, and VEGF in the CRLM sections.^21–24^ The scoring methods are described briefly in Appendix 1. The median HIF-1α, CA-9, and VEGF immunoscores for the cohort was used as a cut off between high and low expression of respective factors. Hence, all scores above the median were classified as high expression, and below and including the median as low. Ki67 expression was quantified by counting the number of stained cells per counted 1000 cells in a high power field (×400).^24^ The scoring methods were pretested and agreed by the pathologists (TV and JW) and first author (PNS) on a sample set of sections. The pathologists were unaware of the clinical outcome and independently assessed the sections. When there was disagreement in scoring, both pathologists assessed the specimens on a multi-head microscope and agreed on the final score.

The distribution of HIF-1α expressing cells within the tumor was studied by scanning immunostained sections of CRLM and the distribution was classified as patchy, central, and peripheral.

It was observed that within CRLM, areas of fibrosis and areas of necrosis had a higher density and a lower density of microvessels, respectively. Hence, we modified the previously described “International consensus on the methodology and criteria of evaluation of angiogenesis in solid tumors”^25^ for the quantification of microvascular density (MVD) in CRLM in order to overcome overestimation or underestimation of microvessels in areas of fibrous septa or areas of tumor necrosis, respectively. Briefly, micro-vessels were counted in all the fields within the tumor using high power magnification and the MVD was calculated by counting the micro-vessels in an area within a 0.25-mm^2^ graticule in a high power field. The mean microvessel count of the highest 5 fields was used to calculate MVD (micro vessels per mm^2^). Our method was comparable to the “hotspot” method of counting microvessels. The intraclass correlation coefficient between the 2 methods was 0.96 (95% confidence interval [CI]: 0.91–0.98) (Siriwardana PN, et al, unpublished data, October 2015).

### Data Analysis

Statistical analyses were performed using Stata/IC 13.1 for Windows, StataCorp (College Station, TX). Due to the small sample size and skewedness, Kruskal–Wallis test was used to compare continuous data such as age, MVD, and Ki67 count between the 2 groups. The Fisher exact test was used to assess the association between categorical variables such as HIF-1α, CA-9, and VEGF scores. Missing data were excluded from the analysis. Interobserver variability of immunoscoring of HIF-1α, CA-9, and VEGF expression was assessed using kappa statistics. The kappa value was interpreted as: <0.2, poor; 0.21 to 0.40, fair; 0.41 to 0.60, moderate; 0.61 to 0.80, good; and 0.81 to 1.00, excellent. Kaplan–Meier plot and Cox regression-based test for equality of survival curves were used to summarize the overall survival (OS) experience of the patients by CRLM group.

## RESULTS

### Tumor Morphology

**FIGURE 2:**
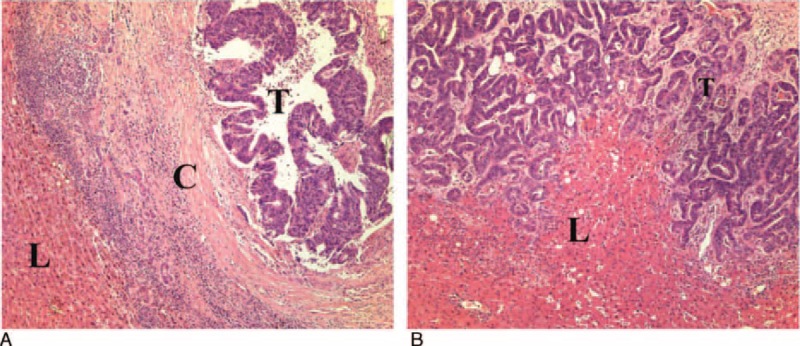
Morphology of colorectal liver metastases (CRLM). H&E staining of CRLM showing (A) encapsulated CRLM with fibrotic or desmoplastic layer (C) between the metastasis (T) and the liver parenchyma (L) with large, complex, cribriform glands. (B) Infiltrative CRLM with no fibrous or desmoplastic layer in the interface with an “infiltrative” interface had small, closely packed and individually arranged rounded glands. C = fibrous capsule, H&E = hematoxylin and eosin, L = liver, T = tumor.

There were 2 distinct types of CRLM as regard to the morphological appearance of the tumor to normal liver parenchyma interface. The first type had a fibrotic or desmoplastic layer between the metastasis and the liver parenchyma. There was a lymphocytic infiltrate and bile ductules within the layer of desmoplasia. The tumor margin was intact. The glands were large, complex, and cribriform (Figure 2A). These were named as “encapsulated CRLM.”

In the second type, which were named as “infiltrative CRLM,” the metastasis was infiltrating the liver sinusoids with no desmoplasia or lymphocytic infiltrate in the CRLM-liver interface. The glands were small, closely packed, individually arranged, and rounded (Figure 2B). Encapsulated CRLM had more necrosis compared with infiltrative CRLM, but this was not quantified.

If in case 2 types of interfaces were present in separate areas of the tumor margin, the metastasis was classified as 1 or the other type if 75% or more of the interface fitted the above description. However, if both types were present and if any 1 type was more than 25% of the interface, it was classified as a mixed type.^26^ Thirteen (43%) patients had a “fibrotic or desmoplastic” interface, 13 (43%) had an “infiltrative” interface, and 3 (10%) had a mixed interface. The tumors with a mixed interface were excluded from further analysis. One CRLM (4%) which had an intact tumor margin but did not have a fibrotic interface, did not fit into the above classification, and was not analyzed further.

**FIGURE 3:**
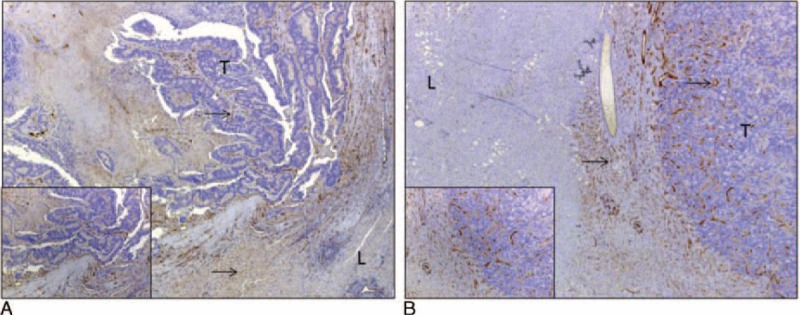
Microvascular pattern of colorectal liver metastases (CRLM). CD31 staining characteristics and (A) encapsulated CRLM showing scanty micro vessels confined to the stroma supporting the glands (×4 and ×10). (B) Infiltrative CRLM showing vessels distributed in the interglandular fibrovascular connective tissue (×4 and ×10). (A) and (B) both show the expression of CD31 in peritumoral sinusoids (arrows pointing at microvessels). CD31 = cluster of differentiation 31, L = liver, T = tumor.

In encapsulated CRLM, CD31 immunostaining revealed the micro vessels to be scanty and confined to a stroma surrounding the glands (Figure 3A). In infiltrative CRLM, vessels were distributed in the interglandular fibrovascular connective tissue (Figure 3B). This pattern was most noticeable at the periphery of the lesions near the interface.

There was a high interobserver agreement between pathologists in correctly identifying these 2 morphologies (kappa = 0.88, 95% CI: 0.73–1.00).

The clinicopathological characteristics of the patients who had encapsulated CRLM and infiltrative CRLM are shown in Table 2. The groups were similar with regard to age, sex, primary tumor site, Duke's stage, differentiation of primary, chemotherapy for primary, temporal relationship of developing liver metastases (synchronous/metachronous) to the presentation of primary, differentiation of CRLM, tumor burden, and involvement of the resection margin. None of the patients had extra hepatic metastatic disease.

**TABLE 2:**
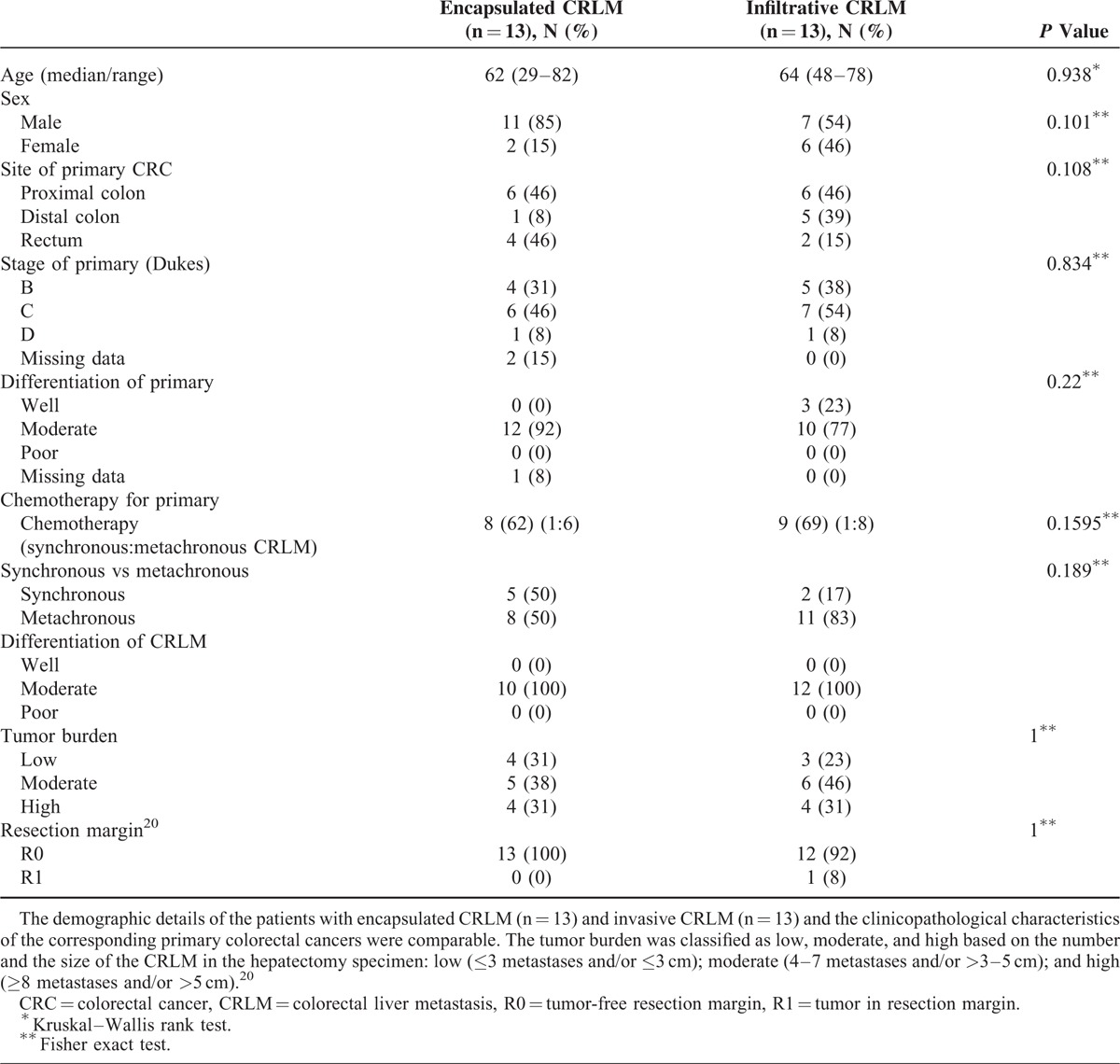
Clinicopathological Characteristics of the Primary Colorectal Cancer and the Colorectal Liver Metastases

### Overall Survival

Long-term survival data were available in 25 of the 26 patients.

The median period of follow-up was 9.5 years (115 months—ranging from 6 to 182 months). Eleven (42%) of the 26 patients died, 14 (54%) are alive, and 1 patient was lost to follow-up. The 5-year OS rate following surgical resection for CRLM was 69%. The 5-year survival rate in the encapsulated CRLM was 11/13—85% (95% CI: 51–96) but in the infiltrative group was 54% (95% CI: 19–70; Figure 4). The survival curves differed considerably between encapsulated and infiltrative CRLM (relative hazard [RH]: 0.58, *P* = 0.057, Cox regression) with a more favorable prognosis in patients with encapsulated CRLM. The 5-year survival rates for the encapsulated and invasive CRLMs were 84% (95% CI: 51–96) and 46% (95% CI: 19–70), respectively, and the difference was significantly different (RH: 0.518, *P* = 0.044, Cox regression).

**FIGURE 4:**
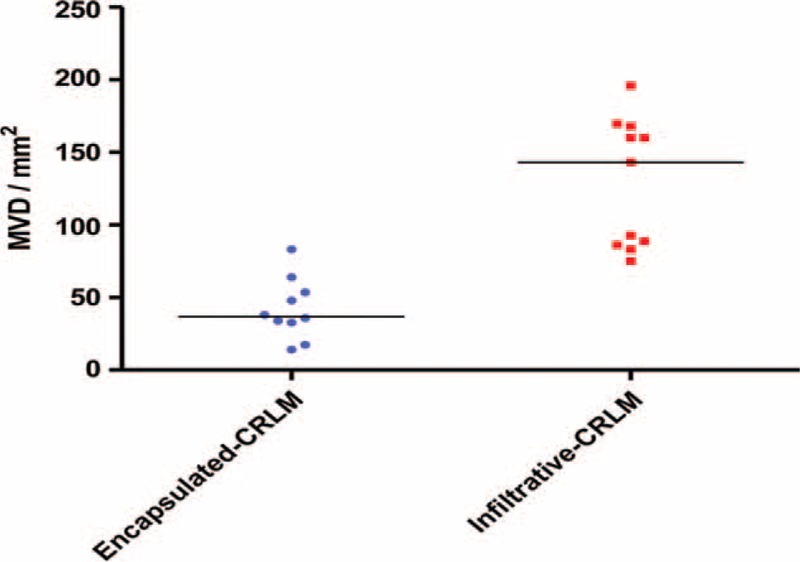
Kaplan–Meier plot showing the overall survival and 5 survival of patients with encapsulated CRLM and infiltrative CRLM. The overall survival curves differed considerably between encapsulated and infiltrative CRLM (relative hazard [RH]: 0.58, *P* = 0.057, Cox regression). The 5-y survival rate in the encapsulated CRLM and infiltrative CRLM was 84% (95% CI: 51–96) and 46% (95% CI: 19–70), respectively. The 5-y survival curves differed significantly (RH: 0.518, *P* = 0.044, Cox regression). CI = confidence interval, CRLM = colorectal liver metastasis.

### Tumor Hypoxia

We examined the relationship between the expression of hypoxic factors, HIF-1α and CA-9, and the morphology of the CRLM. Immunohistochemistry scoring was not possible in 2 cases, 1 from each group, due to extensive necrosis within the tumor.

There was a significantly higher expression of hypoxia regulated factors in encapsulated CRLM compared with infiltrative CRLM. The median HIF-1α score for the study population was 3 (range 0–4). Fifty-eight percent (7/12) of patients with encapsulated CRLM and 8% (1/12) of patients with infiltrative CRLM had a high expression of HIF-1α (Fisher *P* = 0.03). There was no evidence of a difference between the distribution patterns of HIF-1α within the CRLM in the 2 groups (Fisher *P* = 0.22). When both groups were combined, the distribution pattern of HIF-1α was patchy in 16 (73%), central in 5 (23%) and only in 1 (4%) of the metastases in the invasive front.

The median CA9 score for the study population was 2 (range 0–3). Forty-two percent (5/12) of patients with encapsulated CRLM had a high CA9 score. None of the patients with infiltrative CRLM, however, had a high CA9 score expression (Fisher *P* = 0.039).

### VEGF Expression

The VEGF expression pattern differed significantly between the 2 morphological appearances of CRLM. The median VEGF score for the study population was 3 (range 0–6). Ninety-two percent (11/12) of patients with encapsulated CRLM and 25% (3/12) of patients with infiltrative CRLM had high VEGF expression (Fisher *P* = 0.02).

The interobserver agreement (kappa) for immunoscoring of HIF-1α, CA-9, and VEGF was high and the kappa was, respectively (kappa [95% confidence interval (CI)]), 0.82 (0.62–1.00), 0.72 (0.48–0.97), and 0.76 (0.56–0.97).

Table 3 summarizes the expression of HIF-1α, CA-9, and VEGF, in encapsulated CRLM and infiltrative CRLM.

**TABLE 3:**
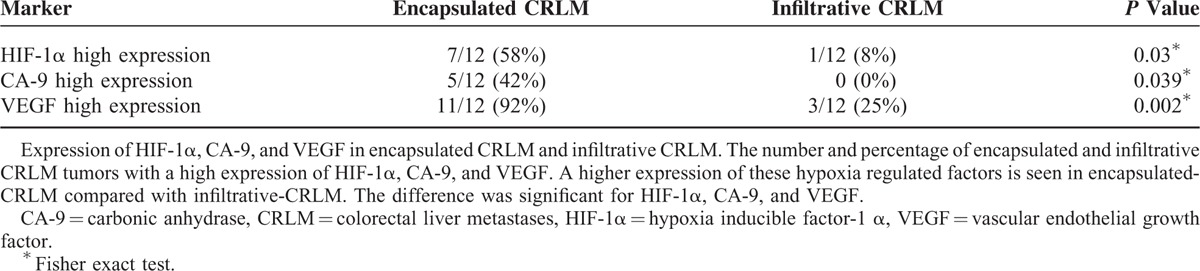
Expression of HIF-1α, CA-9, and VEGF in Encapsulated CRLM and Infiltrative CRLM

### Microvascular Density

**FIGURE 5:**
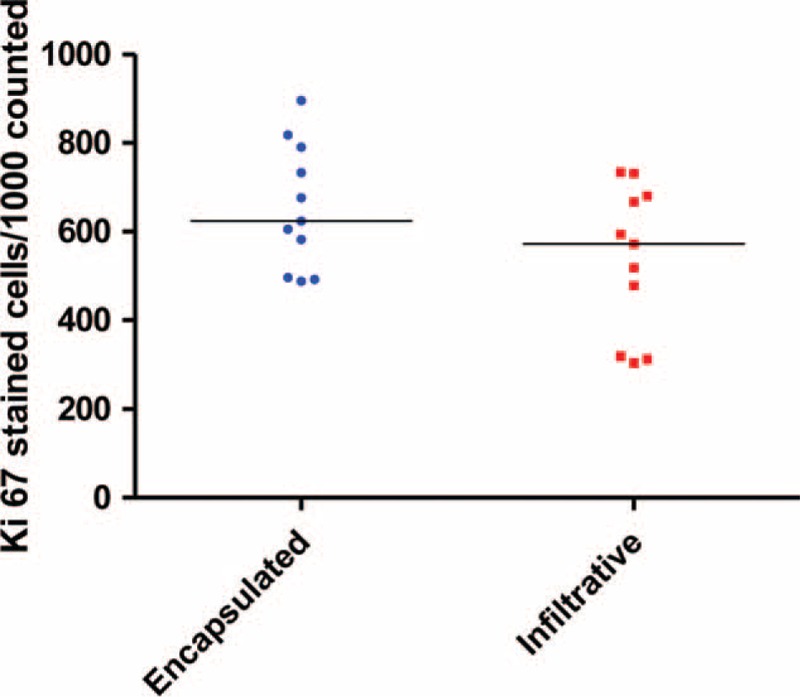
Microvascular density of CRLM as determined by CD31 staining. Difference in the median microvascular density of encapsulated and infiltrative CRLM 37 per mm^2^ (range 14–83) vs 143 per mm^2^ (range 75–196). Kruskal–Wallis equality of populations rank test *P* < 0.001. CRLM = colorectal liver metastasis.

MVD was analyzed for 22 patients. Four cases, 2 from each morphological type, were excluded from the analysis as sections contained inadequate tissue for microvessel counting due to tumor necrosis. CD31 was expressed in the cytoplasm of endothelial cells which lines the intratumoral microvessels and the peritumoral hepatic sinusoids (Figure 5). Encapsulated CRLM had a lower microvessel density than the infiltrative CRLM. The median MVD of encapsulated CRLM and infiltrative CRLM were 37 per mm^2^ (range 14–83) and 143 per mm^2^ (range 75–196), respectively (Kruskal–Wallis equality of populations rank test *P* < 0.001).

### Tumor Proliferation

**FIGURE 6:**
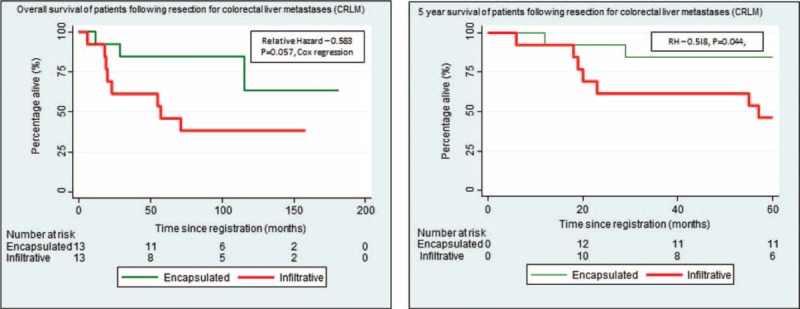
Tumor proliferation of CRLM as determined by Ki67 staining. The median Ki67 stained cells per 1000 counted cells in encapsulated and infiltrative CRLM (624 [range 488–896] vs 572 [304–734], *P* = 0.14; Kruskal–Wallis equality of populations rank test *P* = 0.14. Data available only for 11 patients each in the encapsulated and infiltrative CRLM. CRLM =colorectal liver metastasis.

The tumor proliferative fraction median (Ki67 count) was not different between the 2 groups (624 [range 488–896] vs 572 [304–734]; *P* = 0.14, Kruskal–Wallis rank test; Figure 6).

## DISCUSSION

This study has analyzed the morphology of CRLM and correlated this with prognosis in patients resected without neoadjuvant chemotherapy. The morphological types were found to be distinct in the majority of cases and the evaluation of tumor type to be reproducible. The study then proceeded to correlate the morphology with markers of tumor hypoxia and angiogenesis. In addition to the significant difference in survival, this is the first study to demonstrate a difference in expression of HIF-1α, CA-9, and VEGF, and proliferation index between the morphological types of CRLM. When comparing the CRLM–liver interface, encapsulated CRLM were similar to the previously described fibrous pseudocapsule and/or “desmoplastic growth pattern,” and infiltrative CRLM were similar to those without a fibrous capsule and/or “replacement growth pattern.”^5,7,8^ The prognostic significance of the pseudocapsule of CRLM has been studied by at least 3 previous groups.^5–7^ The 5-year recurrence rate was significantly lower in patients with CRLM with a pseudocapsule in all studies. Only Okano et al, however, compared the 5-year OS in the 2 groups and showed a significantly better OS for those with metastases with a capsule (encapsulated vs no capsule: 75% vs 31%).^5^ Our 5-year OS for the corresponding groups, despite no chemotherapy, was considerably higher.

Resection of CRLM is associated with a 5-year survival of up to 32% to 65%.^27,28^ In this study, the 5-year OS rate for the patients with the 2 types of CRLM was higher than published figures. A study from our group on the outcome of patients undergoing resection of colorectal metastases by Hewes et al^29^ demonstrated a high survival rate in patients who did not have chemotherapy prior to resection of CRLM. The authors hypothesized that chemotherapy was not given to this patient group, perhaps due to a good prognosis being anticipated preoperatively, on the merits of a high proportion of patients having metachronous lesions and a significantly fewer number of hepatic metastases in this group. However, the benefit of neoadjuvant chemotherapy for resectable CRLM remains a matter of debate.^30^

Despite the higher expression of HIF-1α, CA-9, and VEGF in the encapsulated CRLM, the OS of this patient group was significantly better than those with infiltrative tumors. The fibrotic layer at the CRLM–tumor interface functioning as a barrier to metastasis, restraining growth and further spread of CRLM may explain the survival advantage of this group. While, Okano et al^5^ proposed a protective immune inflammatory mechanism of the noncancerous liver against the metastasis giving rise to the pseudocapsule and reducing local recurrence, Lunevicius et al^7^ proposed a mechanical and chemical barrier function of the fibrotic capsule. The fibrotic capsule in hepatocellular carcinoma is known to act as a barrier to the spread of cancer cells.^31^ Hence, the barrier function of encapsulated CRLM may be reducing the aggressiveness and metastatic growth, which would otherwise result due to the increased expression and activation of the HIF-1α pathway.

Tumors cannot grow in vivo for more than 1 to 2 mm^3^ without a blood supply for survival. Hence, many tumors, due to their accelerated growth, have areas of hypoxia, contributed to by increased oxygen consumption and aberrant new vessels.^32^ CRLM are known to have a different degree of co-option of vascular network from the adjacent liver parenchyma.^8^ The intratumoral hypoxia in encapsulated tumors which was demonstrated in this study may be caused by the fibrous capsule impeding the recruitment of new vessels from surrounding normal liver parenchyma. The induction of HIF-1α and VEGF could be a result of this hypoxic state. Encapsulated CRLM had a relatively lower MVD compared to infiltrative CRLM. A similar phenomenon has been shown in hepatocelluar carcinoma, where tumors with an intact capsule have a significantly lower MVD compared to those with an incomplete capsule (*P* = 0.01).^33^

A low MVD and higher expression of VEGF was found in encapsulated CRLM and the opposite in the infiltrative CRLM, the mechanism of which is not known. It is contrary to studies assessing the relationship between VEGF and MVD in primary CRC as well as a single study of CRLM in which high expression of VEGF was associated with an increased MVD.^34^ However, Nakamoto et al^35^ who studied the expression of angiogenic factors in primary CRC and CRLM reported a positive correlation between VEGF and MVD in primary CRC but did not find a correlation between VEGF and MVD in liver metastases. The expression of HIF-1α, CA-9, and VEGF in CRLM has been found to be significantly lower than the peritumoral liver parenchyma.^36^ This may be due to CRLM being well perfused by the adjacent rich liver vascular network and also because they are predominantly perfused by hepatic artery.^37^ Hence, we hypothesize that vascularization of CRLM may not be VEGF driven and infiltrative CRLM, which do not have a pseudocapsule, are abundantly vascularized by co-opting the adjacent rich liver vascular network with viability ensured without an increase in expression of VEGF. Some highly vascular cancers have evidence of reduced expression of VEGF. A study in nonsmall cell lung cancer showed that cancers with an angiogenic vascular pattern did not have a significantly higher VEGF.^38^ The encapsulated form, however, could be deprived of this rich vascular supply from the adjacent liver, making them relatively hypoxic with high expression of HIF-1α, CA-9, and VEGF.

Fifteen patients in this study received chemotherapy for their primary CRC. All the metachronous CRLMs were detected after the chemotherapy regimen was completed. Only 2 patients had synchronous CRLM, which would have been affected by the chemotherapy regime for the primary CRC. However, there was an 8- and 12-month period between the completion of the chemotherapy regime for the primary CRC and the resection of the CRLM for these 2 cases. In Rubbia-Brandt et al's study, hepatectomy was performed within a median of 35 days (16–110 days) of completion of chemotherapy for CRLM and the long-term resolution of the chemotherapy-related histological changes within the liver parenchyma and metastases are not known.

Although postoperative histopathological stratification into morphological types help predict the prognosis, the therapeutic value of currently used postoperative chemotherapy regimens for CRLM has not been established.^39^ Preoperative magnetic resonance imaging (MRI) can differentiate encapsulated HCC from nonencapsulated HCC.^40^ Hence, this study provides the evidence to investigate prospectively whether MRI could predict these 2 morphological types of CRLM preoperatively. In addition, PET with hypoxia-specific tracers could be used to differentiate hypoxic encapsulated type from infiltrative type of CRLM.^41^

The main limitation of this study was the small sample size which is due to a combination of 2 factors. These were having to restrict the study period to have a long median follow-up period of almost 10 years and selecting a group of patients who did not have chemotherapy for their CRLM to maintain homogeneity of the study group. Despite the small study population, this proof-of-concept study has demonstrated a nearly significant difference (*P* = 0.057) between the OS and significant difference in the 5-year survival (*P* = 0.044) between the different morphological types of CRLM.

In conclusion, this preliminary study confirms that there are 2 main histological types of CRLM, each with a distinct tumor-liver interface, intratumoral glandular and vascular pattern, and significantly different expression of HIF-1α, VEGF, and MVD. There was a significantly better OS in patients with resected encapsulated CRLM compared to those with infiltrative metastases. There is growing support that the histological type should be included in the histopathology report for resected CRLM.^4^ This study could be used as a benchmark for further multicenter studies as well as analysis of patient groups who have had chemotherapy prior to resection of CRLM, results of which would emphasize the need to include the morphological type in the CRLM histolopathology report. In addition, based on the morphological difference and the oxygenation status of the 2 types of CRLM, imaging techniques could be used to stratify patients into treatment groups.

## APPENDIX 1

For HIF-1α, CA-9, and VEGF, the staining characteristics were categorized into numerical classes. Scores for HIF-1α protein immune reactivity were as follows: 0—no staining; +1—nuclear staining in <1% of cells; +2—nuclear staining in 1% to 10% of cells and/or with weak cytoplasmic staining; +3—nuclear staining in 10% to 50% of cells and/or with distinct cytoplasmic staining; +4—nuclear staining in more than 50% of cells and/or with strong cytoplasmic staining.^21^ Immunoreactivity to CA-9 was categorized as follows: 0—no immunoreactivity; +1—1% to 25% cells immunoreactive, +2—26% to 50%; and +3—>50% cells immunoreactive.^22^ As described previously, cells were considered positive for VEGF if there was unambiguous staining of the membrane or the cytoplasm. A combination of the percentage of positive cells and the intensity was used to quantify VEGF expression. The percentage of positive cells was scored as follows: 0% to 19%—0, 20% to 39%—1, 40% to 59%—2, and 60% to 100%—3. The intensity of VEGF staining was graded as, 1—mild, 2—moderate, and 3—strong. As a benchmark of staining intensity, cells with a staining intensity similar to endothelial cells was classified as grade 2. The percentage score and grade were summed to obtain the VEGF staining score.^23^

## References

[1] Bengtsson G Carlsson G Hafstrom L Natural history of patients with untreated liver metastases from colorectal cancer. *Am J Surg* 1981; 141:586–589. 7223955 10.1016/0002-9610(81)90057-x

[2] Mentha G Majno P Terraz S Treatment strategies for the management of advanced colorectal liver metastases detected synchronously with the primary tumour. *Eur J Surg Oncol* 2007; 33 (Suppl 2):S76–S83. 18006267 10.1016/j.ejso.2007.09.016

[3] Portier G Elias D Bouche O Multicenter randomized trial of adjuvant fluorouracil and folinic acid compared with surgery alone after resection of colorectal liver metastases: FFCD ACHBTH AURC 9002 trial. *J Clin Oncol* 2006; 24:4976–4982. 17075115 10.1200/JCO.2006.06.8353

[4] Knijn N de Ridder JM Punt CJ Histopathological evaluation of resected colorectal cancer liver metastases: what should be done? *Histopathology* 2013; 63:149–156. 23763641 10.1111/his.12124

[5] Okano K Yamamoto J Kosuge T Fibrous pseudocapsule of metastatic liver tumors from colorectal carcinoma clinicopathologic study of 152 first resection cases. *Cancer* 2000; 89:267–275. 10918155

[6] Wiggans MG Shahtahmassebi G Malcolm P Extended pathology reporting of resection specimens of colorectal liver metastases: the significance of a tumour pseudocapsule. *HPB* 2013; 15:687–694. 23458032 10.1111/hpb.12028 PMC3948536

[7] Lunevicius R Nakanishi H Ito S Clinicopathological significance of fibrotic capsule formation around liver metastasis from colorectal cancer. *J Clin Oncol* 2001; 127:193–199. 10.1007/s004320000199 PMC12164968 11260865

[8] Vermeulen PB Colpaert C Salgado R Liver metastases from colorectal adenocarcinomas grow in three patterns with different angiogenesis and desmoplasia. *J Pathol* 2001; 195:336–342. 11673831 10.1002/path.966

[9] van Laarhoven HWM Kaanders JHM Lok J Hypoxia in relation to vasculature and proliferation in liver metastases in patients with colorectal cancer. *Int J Radiat Oncol Biol Phys* 2006; 64:473–482. 16242253 10.1016/j.ijrobp.2005.07.982

[10] Semenza GL Hypoxia-inducible factors: mediators of cancer progression and targets for cancer therapy. *Trends Pharmacol Sci* 2013; 33:207–214. 22398146 10.1016/j.tips.2012.01.005 PMC3437546

[11] Wykoff CC Beasley NJP Watson PH Hypoxia-inducible expression of tumor-associated carbonic anhydrases. *Cancer Res* 2000; 60:7075–7083. 11156414

[12] Déry M-AC Michaud MD Richard DE Hypoxia-inducible factor 1: regulation by hypoxic and non-hypoxic activators. *Int J Biochem Cell Biol* 2005; 37:535–540. 15618010 10.1016/j.biocel.2004.08.012

[13] Shimomura M Hinoi T Kuroda S Overexpression of hypoxia inducible factor-1 alpha is an independent risk factor for recurrence after curative resection of colorectal liver metastases. *Ann Surg Oncol* 2013; 20 (Suppl 3):S527–S536. 23748663 10.1245/s10434-013-2945-2

[14] Brocato J Chervona Y Costa M Molecular responses to hypoxia-inducible factor 1α and beyond. *Mol Pharmacol* 2014; 651–657.6 (May). 24569087 10.1124/mol.113.089623 PMC3990019

[15] Semenza GL Targeting HIF-1 for cancer therapy. *Nat Rev Cancer* 2003; 3:721–732. 13130303 10.1038/nrc1187

[16] Beckner ME Factors promoting tumor angiogenesis. *Cancer Invest* 1999; 17:594–623. 10592767 10.3109/07357909909032845

[17] Gerdes J Schwab U Lemke H Production of a mouse monoclonal antibody reactive with a human nuclear antigen associated with cell proliferation. *Int J Cancer* 1983; 31:13–20. 6339421 10.1002/ijc.2910310104

[18] Govender D Harilal P Dada M CD31 (JC70) expression in plasma cells: an immunohistochemical analysis of reactive and neoplastic plasma cells. *J Clin Pathol* 1997; 50:490–493. 9378815 10.1136/jcp.50.6.490 PMC499981

[19] Rubbia-Brandt L Audard V Sartoretti P Severe hepatic sinusoidal obstruction associated with oxaliplatin-based chemotherapy in patients with metastatic colorectal cancer. *Ann Oncol* 2004; 15:460–466. 14998849 10.1093/annonc/mdh095

[20] Gomez D Morris-Stiff G Toogood GJ Interaction of tumour biology and tumour burden in determining outcome after hepatic resection for colorectal metastases. *HPB* 2010; 12:84–93. 20495651 10.1111/j.1477-2574.2009.00127.x PMC2826665

[21] 1999; Zhong H Marzo AMD Laughner E Overexpression of hypoxia-inducible factor 1α in common human cancers and their metastases cancer research. 59:5830–5835. 10582706

[22] Al-Ahmadie HA Alden D Qin L-X Carbonic anhydrase IX expression in clear cell renal cell carcinoma: an immunohistochemical study comparing 2 antibodies. *Am J Surg Pathol* 2008; 32:377–382. 18300814 10.1097/PAS.0b013e3181570343

[23] Moriyama M Kumagai S Kawashiri S Immunohistochemical study of tumour angiogenesis in oral squamous cell carcinoma. *Oral Oncol* 1997; 33:369–374. 9415339 10.1016/s1368-8375(97)00025-0

[24] Kikuchi M Mikami T Sato T High Ki67, Bax, and thymidylate synthase expression well correlates with response to chemoradiation therapy in locally advanced rectal cancers: proposal of a logistic model for prediction. *Br J Cancer* 2009; 101:116–123. 19491899 10.1038/sj.bjc.6605105 PMC2713712

[25] Vermeulen PB Gasparini G Fox SB Quantification of angiogenesis in solid human tumours: an international consensus on the methodology and criteria of evaluation. *Eur J Cancer (Oxf, Engl 1990)* 1996; 32A:2474–2484. 10.1016/s0959-8049(96)00379-6 9059336

[26] Van den Eynden GG Bird NC Majeed AW The histological growth pattern of colorectal cancer liver metastases has prognostic value. *Clin Exp Metastasis* 2012; 29:541–549. 22476470 10.1007/s10585-012-9469-1

[27] Tomlinson JS Jarnagin WR DeMatteo RP Actual 10-year survival after resection of colorectal liver metastases defines cure. *J Clin Oncol* 2007; 25:4575–4580. 17925551 10.1200/JCO.2007.11.0833

[28] Simmonds PC Primrose JN Colquitt JL Surgical resection of hepatic metastases from colorectal cancer: a systematic review of published studies. *Br J Cancer* 2006; 94:982–999. 16538219 10.1038/sj.bjc.6603033 PMC2361241

[29] Hewes JC Dighe S Morris RW Preoperative chemotherapy and the outcome of liver resection for colorectal metastases. *World J Surg* 2007; 31:353–364.discussion 365-356. 17219289 10.1007/s00268-006-0103-8

[30] Khatri VP Chee KG Petrelli NJ Modern multimodality approach to hepatic colorectal metastases: solutions and controversies. *Surg Oncol* 2007; 16:71–83. 17532622 10.1016/j.suronc.2007.05.001

[31] Iguchi T Aishima S Taketomi A Extracapsular penetration is a new prognostic factor in human hepatocellular carcinoma. *Am J Surg Pathol* 2008; 32:1675–1682. 18769333 10.1097/PAS.0b013e31817a8ed5

[32] Pries AR Cornelissen AJM Sloot A Structural adaptation and heterogeneity of normal and tumor microvascular networks. *PLoS Comput Biol* 2009; 5:e1000394–e11000394. 19478883 10.1371/journal.pcbi.1000394 PMC2682204

[33] Yao DF Wu XH Zhu Y Quantitative analysis of vascular endothelial growth factor, microvascular density and their clinicopathologic features in human hepatocellular carcinoma. *Hepatobiliary Pancreat Dis Int* 2005; 4:220–226. 15908319

[34] Des Guetz G Uzzan B Nicolas P Microvessel density and VEGF expression are prognostic factors in colorectal cancer. Meta-analysis of the literature. *Br J Cancer* 2006; 94:1823–1832. 16773076 10.1038/sj.bjc.6603176 PMC2361355

[35] Nakamoto RH Uetake H Iida S Correlations between cyclooxygenase-2 expression and angiogenic factors in primary tumors and liver metastases in colorectal cancer. *Jpn J Clin Oncol* 2007; 37:679–685. 17846040 10.1093/jjco/hym080

[36] van der Wal GE Gouw ASH Kamps JM Angiogenesis in synchronous and metachronous colorectal liver metastases: the liver as a permissive soil. *Ann Surg* 2012; 255:86–94. 22156924 10.1097/SLA.0b013e318238346a

[37] Taylor I Bennett R Sherriff S The blood supply of colorectal liver metastases. *Br J Cancer* 1978; 38:749–756. 743492 10.1038/bjc.1978.283 PMC2009820

[38] Reinmuth N Payer N Muley T Treatment and outcome of patients with metastatic NSCLC: a retrospective institution analysis of 493 patients. *Respir Res* 2013; 14:139. 24351122 10.1186/1465-9921-14-139 PMC3878319

[39] Power DG Kemeny NE Role of adjuvant therapy after resection of colorectal cancer liver metastases. *J Clin Oncol* 2010; 28:2300–2309. 20368552 10.1200/JCO.2009.26.9340

[40] Chu KK Chan SC Fan ST Radiological prognosticators of hepatocellular carcinoma treated by hepatectomy. *Hepatobiliary Pancreat Dis Int* 2012; 11:612–617. 23232632 10.1016/s1499-3872(12)60232-x

[41] Fleming IN Manavaki R Blower PJ Imaging tumour hypoxia with positron emission tomography. *Br J Cancer* 2015; 112:238–250. 25514380 10.1038/bjc.2014.610 PMC4453462

